# Society Meetings

**Published:** 1895-11

**Authors:** 


### Society Meetings.

The American Association of Obstetricians and Gynecologists held one of its most interesting and satisfactory meetings at Chicago, September 24, 25 and 26, 1895. The attendance of members was large. Numerous papers were read relating to obstetrics, gynecology and abdominal surgery, and the discussions, as usual in this association, were spirited, forceful and instructive.

The president, Dr. J. Henry Carstens, of Detroit, administered the affairs of the association with great discretion, facilitating the transaction of the large amount of business before it with thoroughness and despatch.

The following-named were elected officers for the ensuing year : president, Dr. Joseph Price, of Philadelphia ; vice-presidents, Drs. Albert Hawes Cordier, of Kansas City, and George Sherman Peck, of Youngstown, Ohio ; secretary, Dr. William Warren Potter, of Buffalo ; treasurer, Dr. Xavier Oswald Werder, of Pittsburg ; executive council, Drs. Charles A. L. Reed, of Cincinnati ; James F. W. Ross, of Toronto ; Albert Vander Veer, of Albany ; Lewis S. McMurtry, of Louisville ; and J. Henry Carstens, of Detroit. Seventeen new Fellows were also elected.

The ninth annual meeting was appointed to be held in Richmond, Va., Tuesday, Wednesday and Thursday, September, 15, 16 and 17, 1896. Resolutions of thanks were passed as follows : first, to Dr. J. B. Murphy, chairman of the committee of arrangements, for the efficient manner in which he had provided for the comfort of the Fellows during the meeting and for the delightful yacht sail tendered the Fellows and guests of the association ; second, to the Chicago Gynecological Society, for many courtesies tendered ; and, third, to Messrs. Breslin and Southgate, proprietors of the Auditorium hotel, for the free use of a splendid parlor in which the meeting was held, and for courteous attention to the Fellows who were guests in the house.

---

The ninetieth annual meeting of the medical society of the state of New York will be held at Albany, Tuesday, Wednesday and Thursday, January 28, 29 and 30, 1896.

The president, Dr. Roswell Park, of Buffalo, announces the following business committee : Dr. H. R. Hopkins, of Buffalo, chairman ; Dr. Nathan Jacobson, of Syracuse, and Dr. J. M. Winfield, of Brooklyn. The president requests that members desiring to present papers may promptly notify the chairman of this committee. He also announces that addresses have been promised by Dr. William Pepper, of Philadelphia, and President Eliot, of Harvard University. The latter will speak on the subject of medical education of the future.

---

The Southern Surgical and Gynecological Association will hold its eighth annual meeting at Washington, D. C., November 12, 13

and 14, 1895. The efficient permanent secretary, Dr. W. E. B. Davis, of Birmingham, has prepared a program replete with interesting titles by able authors, which bespeaks an instructive meeting. The following-named are the officers elected for this meeting: president, Dr. Louis McLane Tiffany, Baltimore; vice-president, Dr. Ernest S. Lewis, of New Orleans, and Dr. Manning Simons, of Charleston; treasurer, Dr. Richard Douglas, of Nashville; member of Council for five years, Dr. L. S. McMurtry, of Louisville.

---

The Medico-Legal Society of Jersey City, recently organized, has appointed a committee to secure a building suitable for a clubhouse and large enough to accommodate all the doctors and lawyers in Hudson county. It is probable that a building fronting Van Nest Park will be chosen. An effort will be made by the society to secure the Jersey City law library for the lawyers and the extensive medical library of the late Dr. Beriah A. Watson for the society.

---

The American Public Health Association held a very interesting meeting at Denver recently. Dr. Ernest Wende, health commissioner of Buffalo, was in attendance as a delegate and secured the next meeting for Buffalo. It will undoubtedly be held early in October, 1896, and will be one of the most important meetings of the year.

---

The Medical Association of Central New York held its twenty-eighth annual meeting at Syracuse, on Tuesday, October 15, 1895, under the presidency of Dr. Floyd S. Crego, of Buffalo, with 125 members present. The proceedings and all the papers read will be published in the Journal, beginning with the December issue.

---

The Cleveland Medical Society held a meeting during the afternoon and evening of September 27, 1895, at which were considered sanitary subjects pertaining to the city of Cleveland. The Journal acknowledges an invitation to attend and regrets that it was unable to accept.

---

The Detroit Medical and Library Association held its annual meeting Monday evening, October 7, 1895. A reception to the members of the association was held at the close of the meeting by the president, Dr. Eugene Smith.

